# Engineering and Characterization of Antibacterial Coaxial Nanofiber Membranes for Oil/Water Separation

**DOI:** 10.3390/polym12112597

**Published:** 2020-11-05

**Authors:** Hamouda M. Mousa, Husain Alfadhel, Emad Abouel Nasr

**Affiliations:** 1 Department of Mechanical Engineering, Faculty of Engineering, South Valley University, Qena 83523, Egypt; 2 Department of Mechanical Engineering, University of Portsmouth, Portsmouth PO1 2UP, UK; husainalfadhel2@gmail.com; 3 Department of Industrial Engineering, College of Engineering, King Saud University, Riyadh 11421, Saudi Arabia; eabdelghany@ksu.edu.sa; 4 Department of Mechanical Engineering, Faculty of Engineering, Helwan University, Cairo 11732, Egypt

**Keywords:** polysulfone, cellulose acetate, zinc oxide NPs, composite membrane, coaxial nanofiber, oil/water separation

## Abstract

In the present study, a coaxial nanofiber membrane was developed using the electrospinning technique. The developed membranes were fabricated from hydrophilic cellulose acetate (CA) polymer and hydrophobic polysulfone (PSf) polymer as a core and shell in an alternative way with addition of 0.1 wt.% of ZnO nanoparticles (NPs). The membranes were treated with a 2M NaOH solution to enhance hydrophilicity and thus increase water separation flux. Chemical and physical characterizations were performed, such as Fourier transform infrared (FTIR) spectroscopy, and surface wettability was measured by means of water contact angle (WCA), mechanical properties, surface morphology via field emission scanning electron microscopy (FESEM), transmission electron microscopy (TEM), and microscopy energy dispersive (EDS) mapping and point analysis. The results show higher mechanical properties for the coaxial nanofiber membranes which reached a tensile strength of 7.58 MPa, a Young’s modulus of 0.2 MPa, and 23.4 M J.m^−3^ of toughness. However, treated mebranes show lower mechanical properties (tensile strength of 0.25 MPa, Young’s modulus of 0.01 MPa, and 0.4 M J.m^−3^ of toughness). In addition, the core and shell nanofiber membranes showed a uniform distribution of coaxial nanofibers. Membranes with ZnO NPs showed a porous structure and elimination of nanofibers after treatment due to the formation of nanosheets. Interestingly, membranes changed from hydrophobic to hydrophilic (the WCA changed from 90 ± 8° to 14 ± 2°). Besides that, composite nanofiber membranes with ZnO NPs showed antibacterial activity against *Escherichia coli*. Furthermore, the water flux for the modified membranes was improved by 1.6 times compared to the untreated membranes.

## 1. Introduction 

Wastewater polluted with heavy metals, organic dyes, oils, and other contaminants has a hazardous effect on the human health and environment. Recently, oily wastewater has noticeably increased and become one of many daily global problems due to the lack of water resources and increasing industrial sectors. Wastewater has many sources from industries such as aluminum, textile, steel, petroleum, food, metal finishing, petrochemical, and the leather industry, all of which need water [1]. There are many conventional oil/waters separation methods, such as centrifugation, air flotation, coalescence, gravity separation, etc. These methods have many disadvantages, such as low separation efficiency, time-consuming, and high energy consumptions. In contrast, membrane separation technology is considered as the most prominent technique for oil/water separation possesses. Membrane separation has many advantages, such as steady quality of permeation, relatively low operating cost, and low energy costs [2,3,4,5]. Hence, membrane technology has been extensively applied as a solution for treatment of wastewater that contains oil. It is the most energy-efficient process for removing non-preferred contaminations using membrane-based nanomaterials for water purification [6,7]. 

One of the main challenges of membrane technology is selecting materials that can enhance membrane performance and properties. There are mainly two categories of membrane materials, polymeric and inorganic, i.e., ceramic and composite, that are mainly used in membrane manufacturing as green and environmentally friendly technology in oil/water separation applications [8]. Polysulfone (PSf) membrane is one of the most attractive polymeric materials that has been applied due to its high thermal stability, good mechanical properties, and excellent chemical resistance [9]. However, there are some limitations of the PSf membrane, such as its hydrophobic nature, which limits water permeation through to the opposite side of the membrane (i.e., water flux) unless extra pressure is applied during the separation process. This leads to decreased membrane water flux, poor membrane performance, shortened working life of the membrane, and fouling problems [10]. To overcome these limitations, PSf was blended with other relatively higher hydrophilic polymers to produce high-flux membranes as a solution. Among these polymers, cellulose acetate (CA) is a commonly used polymer in membrane material fabrication due to its characteristics and main features, such as hydrophilic nature, thermal stability, non-toxicity, and chemical resistance [11]. In addition, it has been used in membrane technology with different design patterns, such as solid and core-shell nanofibers [3,12,13]. Embedding inorganic nanomaterials, such as ZnO nanoparticles (NPs), silver (Ag) NPs [14], and graphene, within the polymeric membrane matrix improves the permeation, antifouling, photocatalytic, and antibacterial properties of membranes [15,16]. Besides that, nanocomposite materials provide adequate mechanical strength and thermal stability [17]. Among these materials, zinc oxide nanoparticles (ZnO NPs) have an excellent role in the composite membrane for permeability and surface hydrophilicity, as well as its antibacterial activity [18]. Membranes with antibacterial properties can separate emerging biological pollutants, such as bacteria and viruses in the wastewater and bacteria in the microfiltration process, and this will lead to an efficient disinfection and microbial control [19,20]. As a result, developed membranes with the mentioned properties are needed for more applicable future water treatment industries. 

The electrospinning technique is efficiently used to fabricate polymeric/composite nanofibrous membranes [21,22]. The generated electrospun nanofiber membrane has some features and properties that make it unique, such as higher surface area to volume ratio, nano-sized pore distribution, and adequate mechanical properties of nanofibers [23]. Therefore, electrospinning has the ability to construct and fabricate nanofibrous membranes with different approaches, including core-shell nanofiber, blending nanofiber, needle-free electrospinning [24], and multi-nozzle electrospinning [25]. Coaxial nanofibers by means of the core and shell nozzle are considered as a way to acquire nanofibrous membranes with more additional properties compared to solid nanofiber membranes in oil/water separation [12]. For example, coaxial nanofibers showed adequate mechanical properties and performance in oil rejection from oily wastewater [26]. In addition to a variety of applications in water micro/nano filtration [27], their applications extended to biomedical and pharmaceutical fields [28] that depend on the nanofibers’ topography [29]. 

There have been many efforts to improve membrane porosity and increase water flux. These efforts include the use of hydrophilic polymers and inorganic nanoparticles for developing photocatalytic self-cleaning membranes using nanomaterials such as boron-doped TiO_2_ single-bond andSiO_2_/CoFe_2_O_4_ nanoparticles [30]. In addition, coaxial nanofibers membranes with heterogeneous structures give extra advantages to the membranes compared to their solid counterparts [26]. Herein, an attempt was made to fabricate a novel core-shell nanofiber from two common polymeric membrane materials (PSf and CA) with ZnO NPs via the coaxial electrospinning technique. The core and shell membrane has the advantage of improving hydrophilicity and thus improving the microfiltration process as well as antibacterial properties. The developed membranes were treated by immersion in a 2M NaOH solution to enhance the hydrophilicity properties and water flux. The novel membranes are aimed to be: (1) developed using a core and shell fibers from PSf and CA embedded with ZnO NPs and have antibacterial activity; (2) improve microfiltration performance for oil/water separation application; (3) treated with NaOH solution to improve water flux.

## 2. Materials and Methods

**Figure 1:**
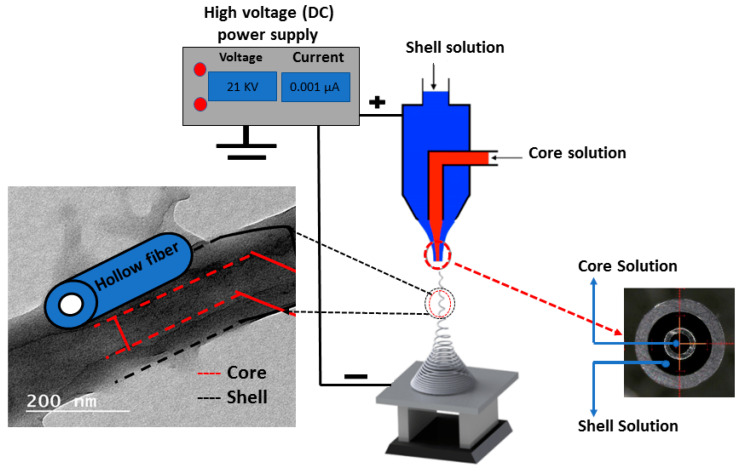
Scheme illustrating the electrospinning technique setup for engineering a coaxial nanofiber membrane.

Figure 1 shows a schematic illustration of the experimental setup for the dual concentric nozzle (coaxial nozzle) via the electrospinning technique (Supplier: NanoNC, Seoul, South Korea). The coaxial nozzle consists of two syringes and the end spinneret has an inner diameter of 0.33 mm coaxially with an outer diameter of 1.07 mm. The solution concentrations of polysulfone (PSf) (average Mn ∼ 50,000 and 40.20 wt.%) PSf was 18% *w*/*v* in N, N-dimethylformamide (DMF), cellulose acetate average (Mn ~ 30,000) (CA) was dissolved in (acetic acid: acetone; 3:1) at 18 wt.% concentrations. The resulting PSf and CA solutions were blended with 0.1 wt.% of zinc oxide nanoparticles (ZnO NPs) for further core and shell fabrication, according to Table 1. Two different solutions of PSf and CA were supplied via the previously described coaxial needle with a syringe pump (Supplier: NanoNC, South Korea). The droplet instantly disintegrated into spinning fibers which were drawn onto a grounded collector. The fabricated membranes were left for 24 h at 40 °C to dry and the residual solvent in the fabricated membrane was removed. Thereafter, the dried electrospun membranes were subject to immersion in sodium hydroxide (2M NaOH) solution for 2 min, followed by rinsing in distilled water and subject to a drying condition at 45 °C for 6 h. All chemicals and reagents used in this work were purchased from Sigma-Aldrich as received.

### 2.1. Membrane Characterizations

#### 2.1.1. Surface Morphology and Material Hydrophilicity

Membrane surface morphology was investigated using field emission scanning electron microscopy (FESEM) and EDS mapping as well as EDS point (QUANTA FEG 250, Thermo Scientific™ Quanta™, Waltham, MA, USA). The developed membranes were exposed to a coating layer from gold at vacuumed condition in an ion sputtering coater at a voltage of 15 to 20 kV. Additionally, the prepared composite membrane was observed with a transmission electron microscope (TEM) (JOEL 2100 PLUS) operated at 200 kV. Deionized water was deposited to the surface of membrane pieces (1 cm × 1 cm) of fibers using a microsyringe (Hamilton Company, Reno, NV, USA). Membrane measurements were performed using the water contact angle (WCA) device (Rame-Hart, Mountain Lakes, NJ, USA). The captured WCA images were extracted using software attached to the device. WCA measurements were calculated for each sample by the mean of three trials measurements. The chemical bonding of the polymeric membranes was characterized with transmittance mode using Fourier transform infrared (FTIR) spectra (Shimadzu FTIR-8400 S, Kyoto, Japan) at wavenumbers from 400 to 4000 cm^−1^ through IR solution software analyzer Version 1.21.

#### 2.1.2. Mechanical Properties and FTIR Analysis

Developed membranes’ mechanical properties through stress and strain were tested with a tensile test machine (model AG-I, Shimadzu, Kyoto, Japan) according to standard test of the thin polymeric sheet ASTM D882-12. The specimen’s size with 5 mm × 1 mm thickness was cut and placed between two grips at speed of 1 mm/min and 30-mm gauge length. Membranes’ mechanical properties, including Young’s modulus, tensile strength, and toughness, were calculated. 

#### 2.1.3. Antibacterial Activity and Oil/Water Separation

The antibacterial activities of composite membranes with ZnO NPs were investigated by zone inhibition methods against *Escherichia coli* (*E. coli*), Gram-negative bacteria as organisms’ model. Muller-Hinton agar was choosing as culture media for *E. coli* (Gram-negative), then agar plates were incubated at 37 °C for 48 h. The filtered paper discs with dimensions of 6.0 mm ± 0.5 mm were used to determine antibacterial test and subject to sterilization using autoclaving for 20 min at 120 °C. The sterile samples were impregnated with different substances (50 mg/mL) at a concentration of 1.5 × 10^8^ CFU/mL. Tetracycline (30 mg/disc) was used as a positive control and the growth inhibition diameter of the developed membrane was then determined. To check bacteria cell viability, 5 mg of the developed membranes ws kept in 5 mL lysogeny broth (LB) with incubated solution exposed to 130 rpm shaking and a temperature of 37 °C for 24 h. Thereafter, *E. coli* with isotonic saline was used as a control without any samples then the optical density of each condition was calculated. All fabricated membranes were evaluated using a laboratory-scale permeation cell unit to separate oil from the water. The exposed area of the membrane in the permeation cell was 19.6 cm^2^, and the water: oil mixture was 3:1 *v:v* using sunflower oil. Membrane water flux was evaluated according to Equation (1) under water gravity conditions.

(1) $$J=\frac{Q}{A\times t}$$

where *J* denotes water flux (m^3^. m^−2^.h^−1^); *Q* denotes water quantity in m^3^; *A* refers to the surface area; *t* is processing time (h).

## 3. Results and Discussion

### 3.1. Membrane Morphology

**Figure 2:**
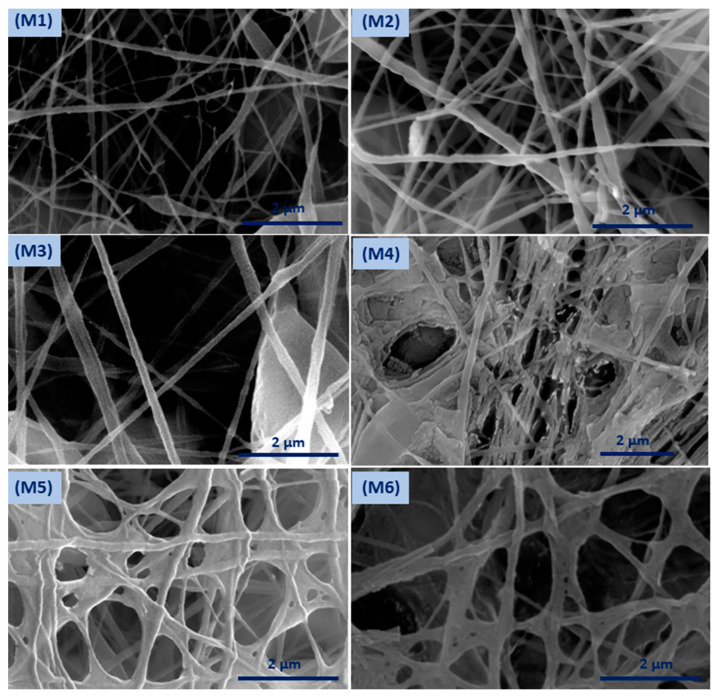
Field emission scanning electron microscopy (FESEM) surface morphology of the developed membranes.

**Figure 3:**
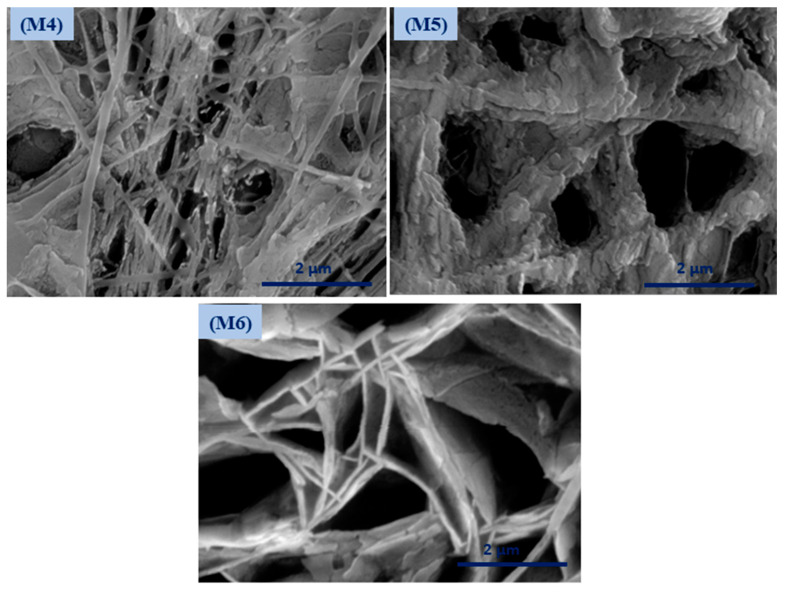
Surface morphology of the modified membrane by NaOH treatment.

**Figure 4:**
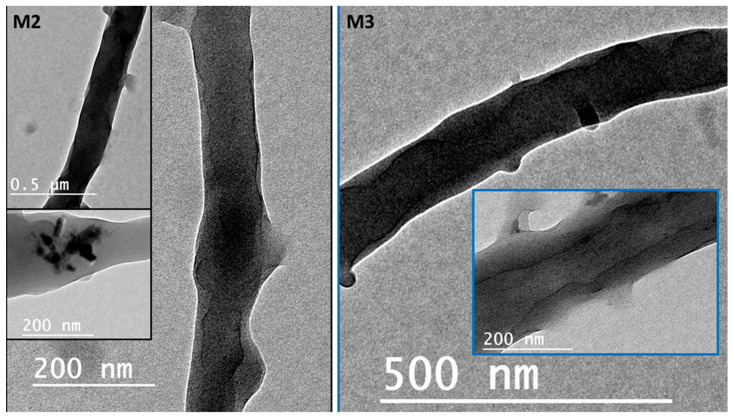
Transmission electron microscopy (TEM) images show the coaxial membranes morphology at different magnifications.

Coaxial electrospinning has been perceived as a productive engineering method to manufacture polymeric and composite nanofibers. Figure 2 shows FESEM images of the fabricated membranes; the electrospun nanofibers orientate randomly and overlap to form a fibrous network structure. The surfaces of the nanofibers demonstrated relatively smooth and beadless nanofibers using optimized solution concentrations related to core and shell **M1** membranes (CA and PSf). The composite ZnO NP nanofibers based on polymer solution at the **M2** and **M3** membranes have a smooth fibrous distribution. Moreover, the treated membranes with NaOH solution are shown in Figure 2 (**M4**–**M6**) and have discontinuous fibers and film formations on the top layer of the membrane that contain ZnO NPs. Figure S1 shows FESEM images (at high resolutions) attributed to the **M3** membrane with core and shell diameters of 102.4 nm and 323.1 nm, respectively. Interestingly, different membrane locations show that coaxial membranes have different morphologies after NaOH treatments, as shown in **M4**–**M6** Figure 3. It is obvious that treatment using NaOH solution changed membranes’ morphology. For example, a polymeric membrane shows the disappearance of nanofibers and is covered with a porous structure. On the other hand, a composite coaxial fiber with shell polymer has thick and porous fibers structure. Furthermore, a coaxial fiber with shell composite of ZnO NPs resulted in novel nanosheets on the outer surface of the composite membrane. This is attributed to the driving force in the hydrothermal environment and the presence of the strong alkaline medium of NaOH [31]. Membranes characterized with TEM images for more understanding of coaxial nanofiber morphology are clear in Figure 4. Moreover, fibers obtained by the coaxial electrospinning of **M2** have a clear image of core and shell fiber formation. TEM images of **M3** have also been observed and show nanoparticles on the shell side attributed to ZnO NPs. 

**Figure 5:**
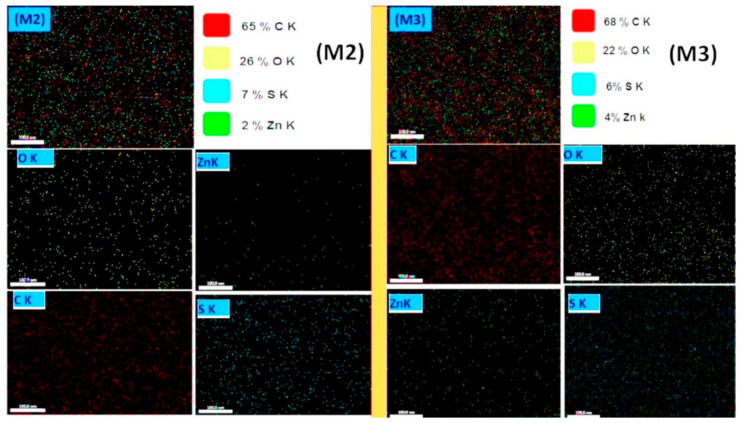
EDS mapping of the developed **M2** and **M3** coaxial nanofiber membranes (scale bar = 100 nm).

The EDS elemental mapping distributions of coaxial nanofiber membranes are shown in Figure 5. Images show homogeneous dispersion of ZnO in the ultrafine membranes fibers regrading the M2 membrane. Besides that, the EDS mapping of the **M2** membrane shows that ZnO NPs were well distributed among the membrane material. The elemental composition of the **M2** membrane shows the elemental composition of 65% C, 26% O, and 2% Zn, in which the core side has ZnO NPs. However, the **M3** membrane shows a higher content of Zn—this is due to the outer shell composite fiber containing CA polymer and ZnO NPs. Figures S2 and S3 show elemental EDS point results of the developed membranes **M1**–**M6** with the presence of all elements of the composite’s membranes. 

### 3.2. Membrane Wettability 

**Figure 6:**
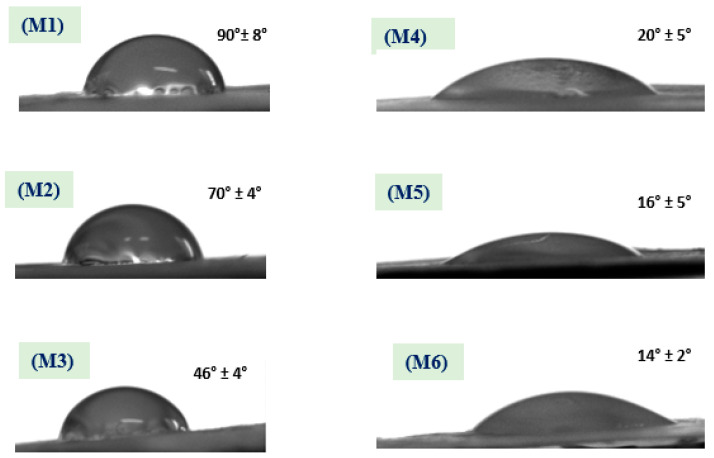
The water contact angle (WCA) of the different membranes shows a decrease in its values after NaOH treatment.

Membrane hydrophilicity plays a vital role in antifouling and permeability; as a result, membranes were evaluated using WCA measurements to assess surface hydrophilic and/or hydrophobic properties. Resulting WCAs of the different membranes (**M1** to **M6**) have the following values: 90° ± 8°, 70° ± 4°, 46° ± 4°, 20° ± 5°, 16° ± 5°, and 14° ± 2°, respectively; the associated WCA images are shown in Figure 6. The resulting WCA of the **M1** membrane shows 90° ± 8°—this decreased to 70° ± 4° after coaxial electrospinning of a shell from CA polymer and coaxial fibers in the **M2** membrane, with similar reported results [32]. Addition of ZnO NPs to PSf and coaxial electrospinning with CA resulted in a lower WCA to 46° ± 4° for the **M3** membrane [33]. Furthermore, surface hydrophilicity of the treated membranes **M4**, **M5**, and **M6** significantly decreased and reached its lowest level (WCA = 14 ± 2°). Overall, surface wettability in terms of WCA decreased from 90 ± 8° for the **M1** membrane to 14 ± 2° after ZnO NPs were embedded in the polymer matrix and NaOH treatment; as shown in Figure 6. Moreover, adding ZnO NPs to shell polymer followed by NaOH treatment has a distinct influence on membrane hydrophilicity. The innovation in the present work is coating membranes with NaOH to alter the membrane’s WCA and water permeability which was achieved by changing the membranes from hydrophobic to hydrophilic. This was also attributed to hydrogen bonds between NaOH layer and water molecules, which can be considered as a positive influence and leads to absorption of water molecules [34].

### 3.3. Membrane Chemical Bonding 

**Figure 7:**
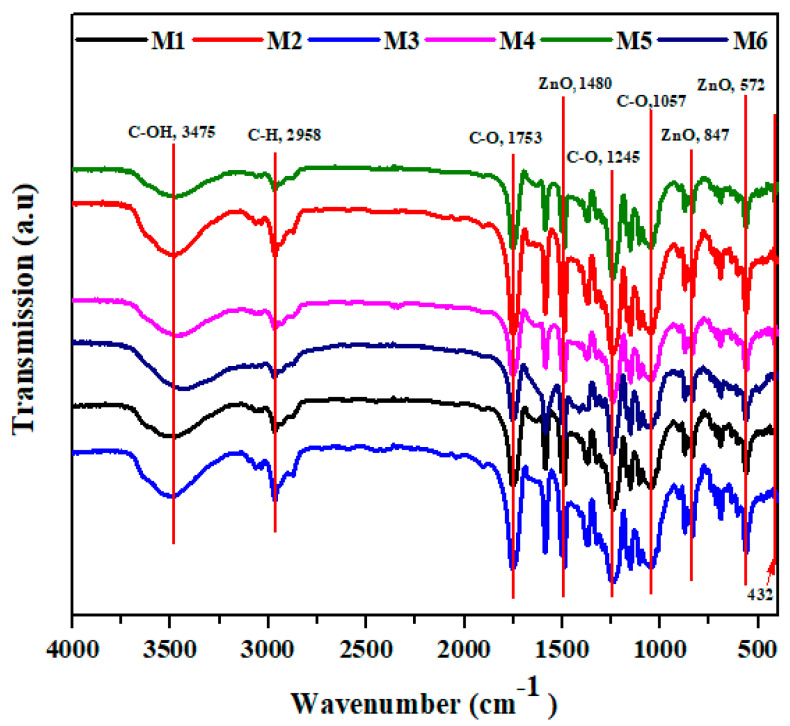
The figure shows the main Fourier transform infrared (FTIR) peak analysis of the developed membranes.

Figure 7 shows the FTIR spectra of all developed membranes. The main peaks at 3475 cm^−1^ are attributed to C–OH vibration; peaks at 753 cm^−1^ are attributed to aromatic ring stretching; peaks at 1480 cm^−1^ are attributed to aromatic semi-ring stretching. Furthermore, the symmetric stretching of S–O appeared at 1057 cm^−1^ that is present in polysulfone polymer [9]. The core and shell (**M1**) membrane shows an absorption band between 400 and 700 cm^−1^ which refers to Si–O–Si. Moreover, bands at 3425 and 1700 cm^−1^ are attributed to the adsorbed water due to the hydrophilic nature of the nanoparticles [35,36,37]. In addition, membranes with CA have characteristic peaks at 3500 cm^−1^ which refer to –OH, at 2963.4 cm^−1^ attributed to –C–H bond, at 1750 cm^−1^ corresponding to C–O, and at 1236 and 1044 cm^−1^ to –C–O– [38]. Furthermore, the **M2** and **M3** membranes have characteristic bands at 460 cm^−1^ that prove the presence of ZnO NPs. The presence of ZnO NP vibration has a peak at 460 and 1749.66 cm^−1^; these peaks are attributed to water adsorbtion on ZnO nanoparticles. Additional peaks of ZnO are observed at 565.44, 827.18, 1384, and 1582.07 cm^−1^ [39]. Moreover, the resulting bands between 3500 and 3000 cm^−1^ are assigned to the stretching band of (-OH) and bending vibration of (H-O-H) in water molecules. Additional absorption of the hydroxyl bands on the surface was observed at 1638 cm^−1^. A similar result was obtained at treated membranes (**M4** to **M6**) with decreasing intensity after treated with sodium hydroxide. 

### 3.4. Membrane Mechanical Properties 

**Figure 8:**
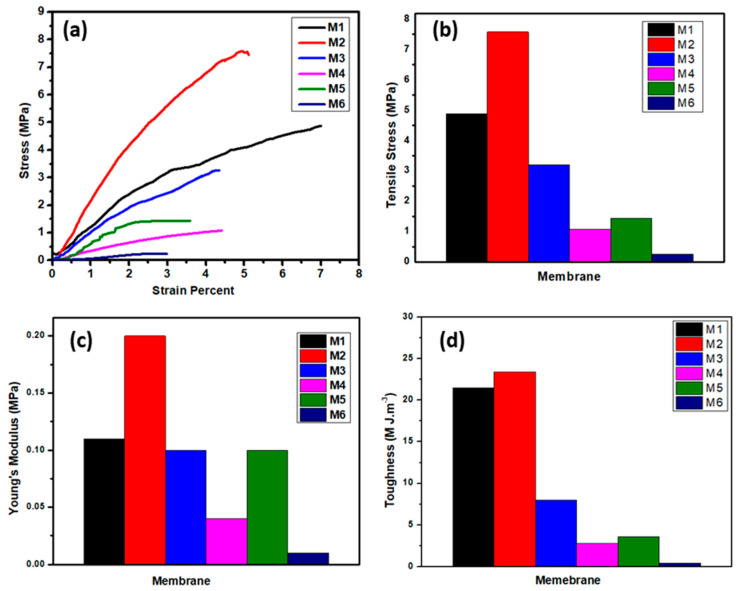
Membranes’ mechanical properties: (**a**) stress–strain curve, (**b**) tensile stress, (**c**) Young’s modulus, and (**d**) toughness of the different membranes.

Figure 8 shows the membranes’ stress and strain curves to evaluate membranes’ mechanical properties which are considered as a key element for the membrane flexibility and durability to overcome the applied pressures of water. The developed membranes were characterized in terms of stress and strain behavior, tensile strength, Young’s modulus, and membrane toughness. The core and shell membrane (**M1**) from PSf and CA polymers shows a higher strain percent, reaching 7%, a tensile strength of 4.89 MPa, and 21.5 M J.m^−3^. Incorporation of ZnO NPs on the **M2** membrane exhibits lower strain behavior and increases the tensile strength value compared to the coaxial **M1** membrane, which is attributed to the inorganic subsitute decreasing the ductility of the composite material [40]. These results show lower ductility and the highest mechnical properties among the membranes (i.e, Young’s modulus, tensile strength, and toughness), as shown in Figure 8a–d. The membrane with PSf core with CA and ZnO shell, **M3**, show the lowest mechnical properties among the developed membranes. On the other hand, the membrane treated with 2M NaOH shows brittleness behavior as indicated from the resulting membrane’s tensile strength, Young’s modulus, and toughness. These findings prove that treatment of polymeric membranes with the alkaline solution of NaOH hinders mechnical properties’ improvement. The trends of the maximum tensile strain and Young’s modulus suggest that the core/shell structure of PSf–CA nanofiber membrane has flexibility and is more elastic than treated membranes with NaOH. 

### 3.5. Antibacterial Activity and Oil Separation

**Figure 9:**
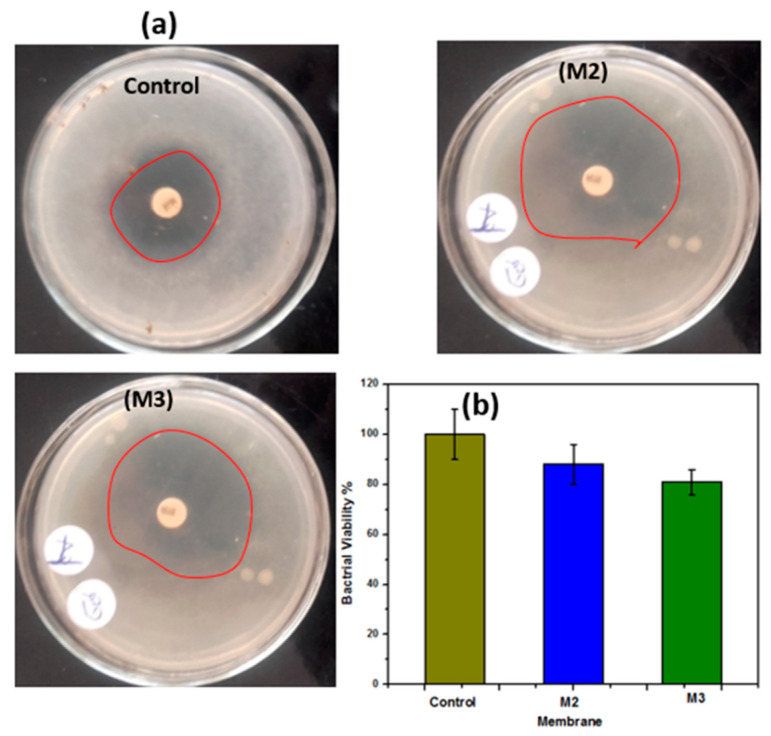
(**a**) Antibacterial properties of the **M2** and **M3** membranes against *E. coli* compared to the control agar plate; (**b**) water flux of the different membranes.

The antibacterial activity of the electrospun membranes was evaluated using *E. coli*. Results show that the manufactured membranes elucidated antimicrobial activity against *E. coli*. Figure 9a shows antibacterial activity against *E. coli* based on zone inhibitors from **M2** and **M3** nanofiber membranes which embeded with ZnO NPs, even in the core or shell. It is suggested that PSf–CA coaxial nanofiber membranes containing ZnO NPs located on the nanofibers’ outer surface hung on the bactrial cell wall and thus resulting in cell membrane incursion [41]. In addition, membranes that contain ZnO resulted in additional antibacterial properties [42]. These results were also confirmed by spectrophotometric analysis. Spectrophotometric analysis is displayed in Figure 9b; the results show that the **M2** and **M3** membranes have the ability to destroy 12% and 19% of *E. coli* bacteria, respectively. The mechanism of antibacterial properties of ZnO NPs is attributed to several reasons which include: (a) interaction of ZnO NPs with the microorganisms and (b) release of antimcrobial ions. As a result of that, damage of bacterial cells happens, and this was also due to the light radiation causing a reactive oxygen species (ROS). The ROS caused a presence of hydroxyl radicals (OH^·^), hydrogen peroxides (H_2_O_2_), and superoxide anion (O_2_) which resulted from the reactive oxygen species [43,44,45,46]. 

**Figure 10:**
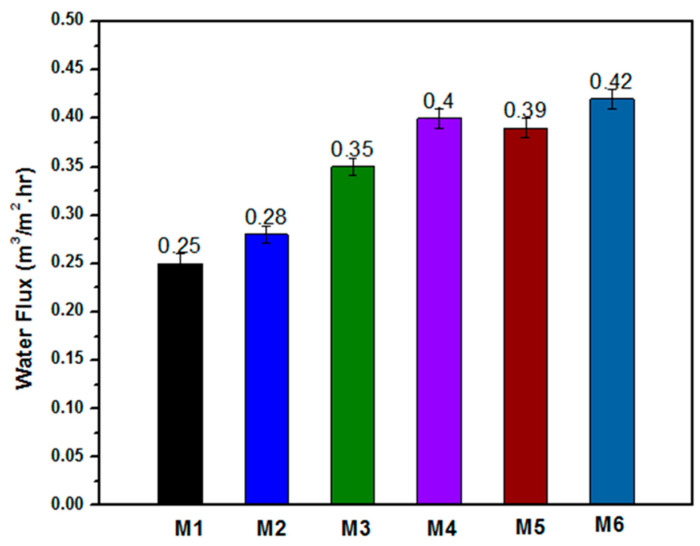
Water flux of the different membranes (**M1** to **M6**); results show water flux increase in treatment membranes.

Membrane water flux was tested to evaluate different electrospun nanofibrous membranes, as shown in Figure 10**.** The developed **M1** membrane has a water flux of 0.25 m^3^.m^−2^.hr^−1^; the **M2** membrane achieved 0.28 m^3^.m^−2^.hr^−1^, and the **M3** membrane resulted in 0.35 m^3^.m^−2^.hr^−1^. After treatment of the membrane with NaOH, the permeability increased relatively, with a high value of water flux among the membrane which resulted in 0.42 m^3^.m^−2^.hr^−1^. The increase in water flux was mainly due to the heat treatment using NaOH solution which mainly made the membrane more hydrophilic, as reported by Lan-Qian Li et al. [47]. A comparison between the present study and related previous studies is summarized in Table 2. Previously developed membranes used the electrospinning technique with membrane mainly composed from PSf and inorganic substitutes, such as NaOH and iron acetate. These membranes exhibited a close water flux, ranging from 0.33 to 0.38 m^3^.m^−2^.h^−1^ after surface coating from polyamide layer deposition. Additionally, the PSf membrane nanofibers had 0.14 m^3^. m^−2^.hr^−1^ of water flux. However, the coaxial membrane shows a higher water flux of 0.25 m^3^. m^−2^.hr^−1^ and a slightly higher WCA due to the hydrophobic nature of the PSf shell. Overall, the coaxial membranes exhibited a higher water flux and, subsequently, higher oil-rejection values compared to the previous studies in similar membrane environmental conditions. These results are accounted to core CA materials and inorganic ZnO NP substitutes which gave an extra advantage of antibacterial effects beside the oil-rejection and mechanical properties.

## 4. Conclusions and Future Perspectives

Novel PSf–CA coaxial nanofiber membranes embedded with ZnO NPs were developed via coaxial nozzle using the electrospinning technique followed by membrane treatment with sodium hydroxide (2M NaOH). The following conclusions are highlighted from this study:

- The results demonstrated smooth and bead-free nanofibers with a unique CA (core) and PSf (shell-ZnO) (shell); the outer diameter was 323.1 nm for PSf (shell-ZnO) and 102.4 nm is the diameter of CA (core).
- Treatment with NaOH layer changed membranes’ WCA from hydrophobic (90 ± 8°) to super hydrophilic (14 ± 2°), and subsequently, membrane water flux increased.
- The permeate flux for coaxial membranes is higher than for solid fiber; moreover, the modified membrane flux was 0.4 m^3^.m^−2^.hr^−1^ while for the unmodified membrane it was 0.25 m^3^.m^−2^.hr^−1^ which is accounted for by NaOH treatment.
- Membrane mechanical properties, in terms of Young’s modulus, tensile strength, and toughness, of the polymeric membranes are higher than those of the NaOH-treated membranes. Furthermore, the membranes have high antibacterial activity and successfully separated water from oil–water wastewater.
- A future prospective study should include an oil/water mixture with the actual content, as the oil/water used in this study was prepared in the laboratory. Future studies should use a real oil/water mixture that includes heavy metals, microorganisms, and dyes to assess the developed membranes. In addition, a pressure-driven cell should be used instead of the gravity-driven cell that was used in the present study as a future filtration device. Besides that, membrane development should include more nanofiber properties, such as flexibility and durability, to consider inspiration at the industrial scale with low cost.

## Figures and Tables

**Table 1:** Fabrication parameters of the developed nanofibrous membranes at room temperature; the table shows the core and shell materials and electrospinning setup conditions.

Membrane	Core	Shell	Remarks
**M1**	PSf	CA	Collector distance 21 cm at 21.7 Kv, feed rate of inner and outer syringe set at 1 mL/h. * This means that ZnO NPs was loaded at 0.1 wt.%.
**M2**	PSf/ZnO NPs *	CA	
**M3**	PSf	CA/ZnO NPs *	
**M4**	¥ Modified M1		¥ This means that membranes were treated by immersion in a 2M NaOH solution for 1 min.
**M5**	¥ Modified M2		
**M6**	¥ Modified M3		

**Table 2:** Present membrane properties compared with reported membranes: membrane materials, water flux, technique, tensile stress, oil type, and membrane features.

Materials	Water Flux (m^3^. m-^2^.hr^−1^)	Technique	WCA	Tensile Stress (MPa)	Young Modulus (MPa)	Toughness (MJ/m^3^)	Antibacterial Properties	Oil Type	Membrane Properties	Ref.
PSf/NaOH	0.33	Electrospinning nanofiber	12°	1.10	**×**	**×**	**×**	Soybean oil	High flux, super hydrophilic.	[48]
(PSf/NaOH) PA layer	0.33		3°	**×**	**×**	**×**	**×**	Soybean oil	Super hydrophilic, high flux.	[49]
PSf	0.14		100°	0.9	3.7	**×**	**×**	Sunflower oil	Hydrophobic, degradation by oil, heat resistance.	[50]
PSf/Iron acetate/PA film	0.38		37°	0.25	7.0	**×**	**×**		High flux, cheap materials, no particles agglomeration.	
**M1**	0.25	Coaxial electrospinning nanofiber	90 ± 8°	4.89	0.11	21.5	**×**		Hydrophobic.	This study
**M2**	0.28		70 ± 4°	7.58	0.2	23.4	**√**		Moderate hydrophilic.	
**M3**	0.35		46 ± 4°	3.2	0.1	8	**√**		Hydrophilic.	
**M4**	0.40		20 ± 5°	1.08	0.04	2.8	**×**		High flux, super hydrophilic.	
**M5**	0.39		16 ± 5°	1.44	0.1	3.6	**√**		High flux, super hydrophilic.	
**M6**	0.42		14 ± 2°	0.25	0.01	0.4	**√**			

## Supplementary Materials

The following are available online at https://www.mdpi.com/2073-4360/12/11/2597/s1, Figure S1: High resolution image of coaxial nanofiber membrane. Figure S2: EDS point analysis of the electrospun co-axial nanofibers membranes before and after surface treatment. Figure S3: EDS point analysis of the electrospun co-axial nanofibers membranes before and after surface treatment in terms of weight and atomic percent.
