# Supplementary material for: Engineering and Characterization of Antibacterial Coaxial Nanofiber Membranes for Oil/Water Separation
Source: Polymers (Basel). 2020 Nov 5;12(11):2597. doi: 10.3390/polym12112597 (PMC7694370; doi:10.3390/polym12112597)
Supplement: Supplementary file 1 [file polymers-12-02597-s001.pdf]

Supporting information of:

Engineering and Characterization of Antibacterial Coaxial Nanofibers Membranes for Oil/Water Separation

Hamouda M. Mousa[1, *], Husain Alfadhel[2], Emad Abouel Nasr[3,4]

[1]Department of Mechanical Engineering, Faculty of Engineering, South Valley University, Qena 83523, Egypt.

[2]Department of Mechanical Engineering, University of Portsmouth, UK.

[3]Department of Industrial Engineering, College of Engineering, King Saud University, Riyadh 11421, Saudi Arabia.

[4]Department of Mechanical Engineering, Faculty of Engineering, Helwan University, Cairo 11732, Egypt.

* Author to whom correspondence should be addressed.

E-Mail: H. M. Mousa (hmousa@eng.svu.edu.eg)

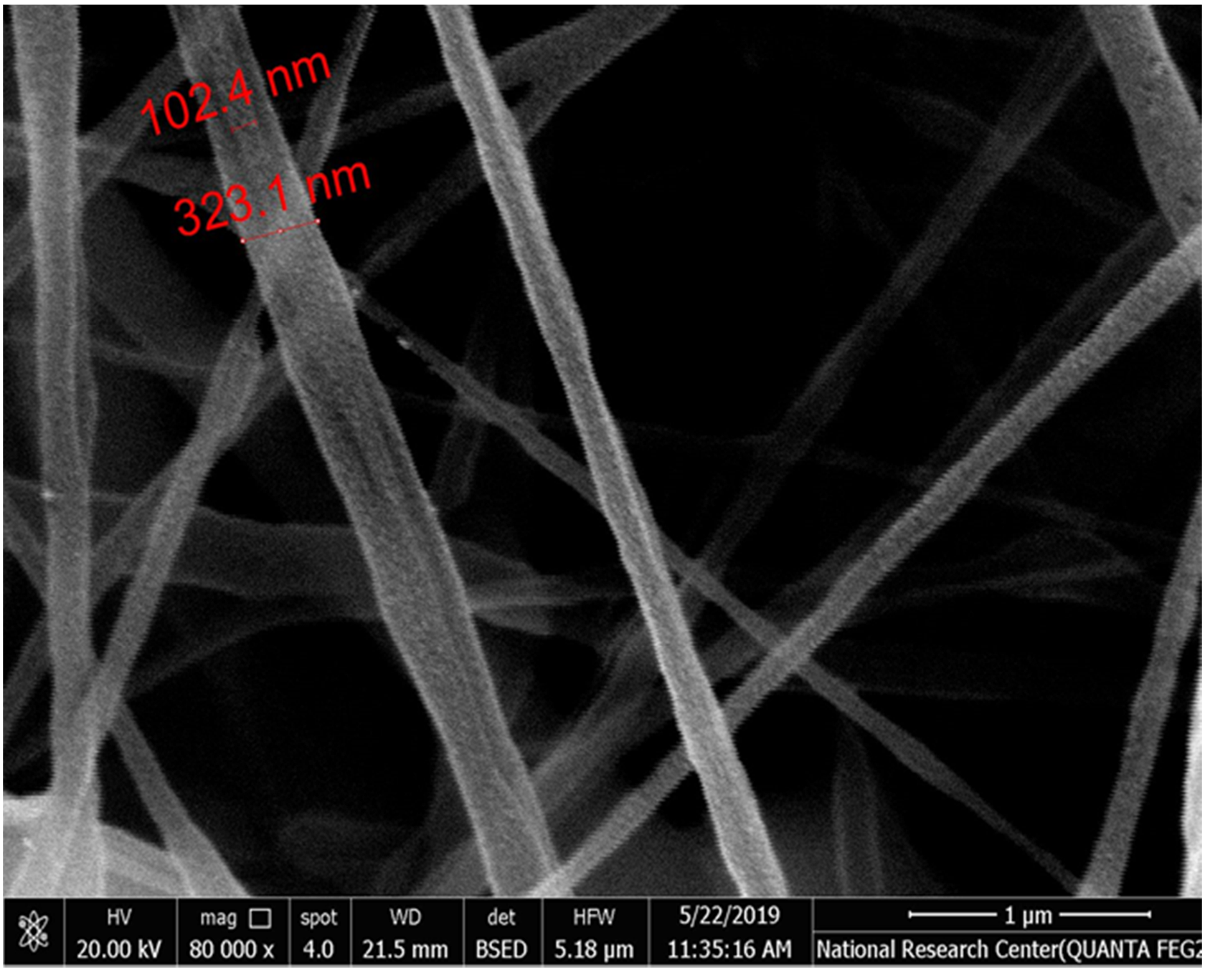

Fig.S1 high resolution image of coaxial nanofiber membrane.

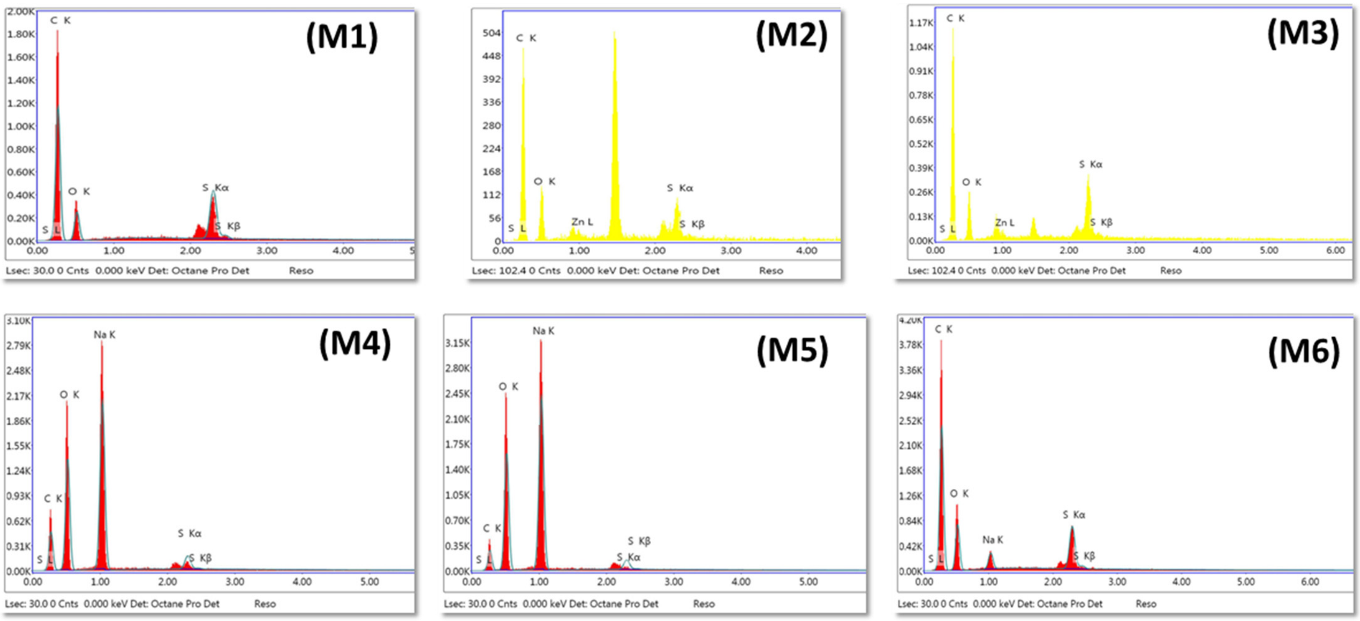

Fig.S2 EDS point analysis of the electrospun co-axial nanofibers membranes before and after surface treatment.

(M1)

Element	Weight %	Atomic %	Net Int.	Error %
C K	71.66	78.83	282.63	7.86
O K	22.93	18.94	64.05	12.52
S K	5.41	2.23	148.31	3.03

(M2)

Element	Weight %	Atomic %	Net Int.	Net Int. Error
C K	65.05	74.89	19.9	0.03
O K	25.48	22.03	6.7	0.05
S K	4.91	2.12	11.3	0.05
ZnK	4.56	0.96	1.5	0.18

(M3)

Element	Weight %	Atomic %	Net Int.	Net Int. Error
C K	68.64	78.45	49.6	0.02
O K	21.19	18.18	12.9	0.03
S K	5.68	2.43	32.2	0.03
ZnK	4.49	0.94	3.7	0.17

(M4)

Element	Weight %	Atomic %	Net Int.	Error %
C K	28.74	38.52	117.07	10.1
O K	38.83	39.07	358.04	9.21
NaK	30.9	21.64	595.94	7.35
S K	1.53	0.77	56.33	9.13

(M5)

Element	Weight %	Atomic %	Net Int.	Error %
C K	20.32	28.61	68.59	11.06
O K	40.65	42.97	419.91	8.52
NaK	37.65	27.69	681.66	7.2
S K	1.37	0.72	44.98	9.69

(M6)

Element	Weight %	Atomic %	Net Int.	Error %
C K	64.7	72.7	583.45	7.43
O K	28.27	23.85	198.95	11
NaK	2.94	1.73	77.53	9.51
S K	4.09	1.72	248.88	2.77

Fig.S3 EDS point analysis of the electrospun co-axial nanofibers membranes before and after surface treatment in terms of weight and atomic percent.
